# Dietary Nisin Modulates the Gastrointestinal Microbial Ecology and Enhances Growth Performance of the Broiler Chickens

**DOI:** 10.1371/journal.pone.0085347

**Published:** 2013-12-20

**Authors:** Damian Józefiak, Bartosz Kierończyk, Jerzy Juśkiewicz, Zenon Zduńczyk, Mateusz Rawski, Jakub Długosz, Anna Sip, Ole Højberg

**Affiliations:** 1 Department of Animal Nutrition and Feed Management, Poznań University of Life Sciences, Poznań, Poland; 2 Institute of Animal Reproduction and Food Research of Polish Academy of Sciences, Olsztyn, Poland; 3 Department of Biotechnology and Food Microbiology, Poznań University of Life Sciences, Poznań, Poland; 4 Department of Animal Science, Arhus University, Tjele, Denmark; German Institute of Human Nutrition Potsdam-Rehbrücke, Germany

## Abstract

Due to antimicrobial properties, nisin is one of the most commonly used and investigated bacteriocins for food preservation. Surprisingly, nisin has had limited use in animal feed as well as there are only few reports on its influence on microbial ecology of the gastrointestinal tract (GIT). The present study therefore aimed at investigating effects of dietary nisin on broiler chicken GIT microbial ecology and performance in comparison to salinomycin, the widely used ionophore coccidiostat. In total, 720 one-day-old male Ross 308 chicks were randomly distributed to six experimental groups. The positive control (PC) diet was supplemented with salinomycin (60 mg/kg). The nisin (NI) diets were supplemented with increasing levels (100, 300, 900 and 2700 IU nisin/g, respectively) of the bacteriocin. The negative control (NC) diet contained no additives. At slaughter (35 days of age), activity of specific bacterial enzymes (α- and β-glucosidases, α-galactosidases and β-glucuronidase) in crop, ileum and caeca were significantly higher (*P*<0.05) in the NC group, and nisin supplementation decreased the enzyme activities to levels observed for the PC group. A similar inhibitory influence on bacterial activity was reflected in the levels of short-chain fatty acids (SCFA) and putrefactive SCFA (PSCFA) in digesta from crop and ileum; no effect was observed in caeca. Counts of *Bacteroides* and *Enterobacteriacae* in ileum digesta were significantly (*P*<0.001) decreased by nisin and salinomycin, but no effects were observed on the counts of *Clostridium perfringens*, *Lactobacillus*/*Enterococcus* and total bacteria. Like salinomycin, nisin supplementation improved broiler growth performance in a dose-dependent manner; compared to the NC group, the body weight gain of the NI_900_ and NI_2700_ groups was improved by 4.7 and 8.7%, respectively. Our findings suggest that dietary nisin exerts a mode of action similar to salinomycin and could be considered as a dietary supplement for broiler chickens.

## Introduction

Lactic acid bacteria (LAB) of the genera *Lactococcus*, *Streptococcus, Pediococcus, Leuconostoc, Lactobacillus* and *Carnobacterium* are the most commonly used starter cultures in food industry. Their mode of action is mainly based on the antimicrobial effect of lactic acid and pH reduction in combination, but certain LAB are capable of producing different types of bacteriocins, which are small peptides, lethal to bacteria other than the producing strain, but most often with a rather narrow target spectrum in comparison to antibiotics. 

Bacteriocins are found in the gastrointestinal tract (GIT) of animals, in soil as well as in food, e.g. fermented milk products, cheese, or meat. The widespread use of bacteriocins in food industry is due to their antimicrobial activity against key Gram-positive pathogens of food-borne diseases, such as *Listeria monocytogenes* or *Staphylococcus aureus*. In contrast, there is so far only limited information on their usage and efficacy in feed industry [1]. 

In a series of our studies, we have recently demonstrated significant effects of bacteriocin - divercin AS7 - on the broiler chicken GIT microbiota and fermentation status as well as bird performance [2-5]. Moreover, other studies have shown dietary efficacy of other bacteriocins in broiler chickens [6,7]. However, to our knowledge there are no available data on *in vivo* usage and effects of nisin; probably the most explored and commonly used bacteriocin in many types of human foodstuffs. 

Nisin is a 34 amino acid residues peptide, with a molecular mass of 3.5 kDa and classified as a class-Ia bacteriocin or lantibiotic [8]. Its usage in food is approved by the European Union with the assigned E-number E234 (EEC, 1983). In USA it has been granted GRAS (Generally Recognized As Safe; notice no. GRN 000065) status by the United States Food and Drug Administration. However, nisin is not included in the European Union Register of Feed additives (EU 1831/2003). Thus, even though this bacteriocin appears in many dairy products and active packing systems, its usage in animal nutrition is still forbidden. 

A recent study by Udompijitkul et al. [9] indicated that nisin exerts strong antimicrobial activity in a meat model system against different isolates of *Clostridium perfringens*; an important poultry pathogen. Earlier studies conducted by Bernbom et al. [10] have shown that application of nisin-producing *Lactococcus lactis* strain to rat diets, affected the composition of the intestinal microbiota by increasing *Bifidobacterium* numbers and suppressing the enterococci/streptococci population. The aim of the present study was therefore to evaluate the influence of dietary nisin on broiler chicken performance and GIT microbial ecology.

## Material and Methods

### Ethics statement

This study was carried out in strict accordance with the recommendations of the National Ethic Commission (Warsaw, Poland). All procedures and experiments complied with the guidelines and were approved by the Local Ethic Commission of the Poznań University of Life Sciences (Poznań, Poland) with respect to animal experimentation and care of animals under study, and all efforts were made to minimize suffering.

### Birds and Housing

In total, 720 one-day-old male Ross 308 chicks were randomly distributed to 6 experimental groups using 12 replicate pens per treatment and 10 birds per pen. The broiler chickens were kept in floor pens (1.2 × 0.8 m) over a production period of 35 d. The birds were given 23 h of light and 1 h of dark during the first week and then 19 h of light and 5 h of dark from d 7 to 21. From 22 to 42 d of age, there was 23 h of light and 1 h of dark.

### Diets and Feeding Program

The composition of the experimental diet is shown in Table 1. The nisin activity was expressed in international activity unit (IU). The diet was formulated to stimulate the proliferation of *C. perfringens* by use of viscous cereals (barley/wheat), animal fats (beef tallow/pig lard), and fishmeal [11-13]. The diets were prepared in mash form; all raw materials were ground by disc mill (Skiold A/S, Denmark) at 2.5 mm disc distance, mixed without any heat treatment, and fed *ad libitum* to the birds. 

**Table 1:** Composition of the basal diets and its calculated nutritive value.

Ingredients (g/kg)	Diet (1—35 d)
Wheat	326.8
Barley	250.0
Soyabean meal	215.4
Beef tallow	30.0
Pig lard	53.7
Double zero rapeseed meal	60.0
Fish meal	30.0
Monocalcium phosphate	11.0
Mineral-vitamin premix**^*a*^**	5.0
Limestone	4.2
L-Lysine –HCl	2.8
DL-Methionine	2.1
L-Threonine	0.3
Sodium carbonate (Na_2_CO_3_)	1.0
Salt (NaCl)	2.6
Titanium oxide (TiO_2_)**^*b*^**	2.0
Calculated nutritive value (g/kg)	
ME (MJ/kg)	12.95
Crude protein	220.0
Crude fat	100.0
Crude fibre %	34.50
Calcium - Ca %	8.50
Lysine %	13.0
Methionine %	5.5
Methionine + Cystine %	9.3
Threonine %	8.1
P avilaible.	4.20
Analysed composition (g/kg)	
Crude protein	214.0
Crude fibre	39.2
Crude fat	101.2
ME (MJ/kg)	12.89

^a^ Providing the following per kilogram of diet: vitamin A (retinol), 11,166 IU; cholecalciferol, 2,500 IU; vitamin E (alpha tocopherol), 80 mg; menadione, 2.50 mg; cobalamin, 0.02 mg; folic acid, 1.17 mg; choline, 379 mg; D-pantothenic acid, 12.50 mg; riboflavin, 7.0 mg; niacin, 41.67 mg; thiamin, 2.17 mg; D-biotin, 0.18 mg; pyridoxine, 4.0 mg; ethoxyquin,0.09 mg; Mn (MnO_2_), 73 mg; Zn (ZnO), 55 mg; Fe (FeSO_4_), 45 mg; Cu (CuSO_4_), 20 mg;I (CaI_2_O_6_), 0.62 mg; Se (Na_2_SeO_3_), 0.3 mg.

^b^ Replaced corresponding amount of the wheat in each diet, from 30—35 d of broiler age.

The positive control (PC) diet was supplemented with an ionophore coccidiostat (salinomycin, 60 mg/kg); the negative control (NC) diet did not contain any additive. The nisin diets were supplemented with increasing levels of the bacteriocin in liquid form (3.2, 9.7, 20.1 and 87.2 ml/kg, respectively). The concentrations of nisin in the final diets were as follows: 100, 300, 900 and 2700 IU nisin/g (group NI_100_, NI_300_, NI_900_ and NI_2700_, respectively).

### Preparation of nisin and analysis of nisin concentrations

The nisin preparation was prepared according to technology elaborated at the Department of Biotechnology and Food Microbiology, Poznań University of Life Sciences, using the nisin-producing strain *Lactococcus lactis* subsp. *lactis* ATCC11454. The strain was grown into log phase in MRS broth, harvested and stored at -80°C in MRS amended with 20% (vol/vol) glycerol. The strain was propagated twice in MRS broth at 30°C before use (primary cultures).Primary cultivations were performed in batch system using 5-liters fermenters (Bioflo III, New Brunswick). MRS medium without Tween 80 was inoculated with 2% (vol/vol) of an overnight culture of the *L. lactis* strain and incubated anaerobically (in nitrogen-flushed atmospheres) at 30°C for 16h. The suspension was maintained at constant level of pH 6.0 by addition of 5M NaOH. The cells were separated from culture medium by membrane microfiltration. The filtrates were adjusted to pH 6.5 and treated with catalase (300 IU/mL, C-3515, Sigma) to exclude the antimicrobial effects of hydrogen peroxide and heated at 80°C for 10 min in order to inactivate proteases, catalase and kill any residual cells. The filtrate was further concentrated using ultrafiltration. The ultrafiltration process was carried out in an Amicon filtration system (model CH2RSA) equipped in cellulose acetate membranes with cut-off point of 30, and further of 5 kDa. The 10-fold concentrated fluid, containing over 800 mg/liter of nisin (32,000 IU/ml), was used as a feed additive in broiler chicken diets [14].

Nisin concentrations were analysed by a bioassay method based on the method of Matsuzaki et al. [15] as follows. Five millilitres of nutrient broth (Biocorp, Poland) was inoculated with *Staphylococcus aureus* ATCC 25923 and incubated on a shaker (100 strokes/min) at 30°C for 15 h. Fifty microliters of the S. *aureus* cell suspension and 50 μl of the ultra-filtrate of the nisin-producing *L. lactis* culture containing nisin solution were added to 5 ml of fresh medium, and this mixture was incubated under the same conditions. After 12 h (i.e. late log phase), the cell concentration was determined by measuring the OD_600_ using an UV spectrophotometer (model Specord 205, JENA). The samples were diluted so that the OD_600_ was in range of 0.1 to 1.5 absorbance units. The OD_600_ values were observed to be inversely correlated with the amounts of nisin ultra-filtrate added. A calibration curve was made using commercially available nisin standard (Sigma, 1000 IU/mg of solid). Nisin concentration was expressed in milligrams per litre, and a nisin concentration of 1 mg/litre was equivalent to 40 IU/ml [16].

### Data and Sample Collection

The feed intake and body weight of the chickens were measured on days 14, 28 and 35. Mortality was registered throughout the entire experiment. At the end of the trial (35d) from each experimental group, 21 randomly picked chickens (3 chickens from 7 pens) were killed by cervical dislocation. For analyses of the gastrointestinal contents (bacterial enzymes, pH and organic acid concentrations), the contents of crop, ileum and caeca from 3 birds per pen were pooled (7 replicate digesta samples of approx. 10g). The remaining part of the samples was immediately frozen and stored in -80°C for the analysis of organic acids by gas chromatography and the microbiota composition by fluorescent in situ hybridization (FISH) of single bacterial cells. 

### Analysis of pH, Fermentation Products and Bacterial Enzyme Activities

The pH in the pooled digesta samples from crop, ileum and caeca, respectively, was measured immediately after slaughter using a combined glass and reference electrode.

Digesta samples were subjected to short-chain fatty acids (SCFA) analysis, using GC (Shimadzu GC-2010, Kyoto, Japan). The samples (0.5g crop and ileum samples, 0.2g caeca sample) were mixed with 0.2 ml formic acid, diluted with deionised water and centrifuged at 7,211 ×*g* for 10 min. The supernatant was loaded onto a capillary column (SGE BP21, 30 m × 0.53 mm) using an on-column injector. The initial oven temperature was 85°C and was raised to 180°C by 8°C/min and held there for 3 min. The temperatures of the flame ionisation detector and the injection port were 180°C and 85°C, respectively. The sample volume used for GC analysis was 1 μl. The putrefactive SCFA (PSCFA) concentration was calculated as the sum of iso-butyrate, iso-valerate, and valerate concentration in the digesta.

The activity of bacterial enzymes (α- and β-glucosidase, α- and β-galactosidase and β-glucuronidase) in crop, ileum and caeca digesta (in the latter case additionally the activity of α-arabinopyranosidase and β-xylosidase were defined) was measured by the rate of *p*- or o-nitrophenol release from their nitrophenylglucosides, according to the method described elsewhere [17]. The following substrates were used: ρ-nitrophenyl-α-D-glucopyranoside (for α-glucosidase); ρ-nitrophenyl-β-D-glucopyranoside (for β-glucosidase); ρ-nitrophenyl-α-D-galactopyranoside (α-galactosidase); *ο*-nitrophenyl-β-D-galactopyranoside (β-galactosidase); ρ-nitrophenyl-β-D-glucuronide (for β-glucuronidase); ρ-nitrophenyl-α-L-arabinopyranosidase (for α-arabinopyranosidase); ρ-nitrophenyl-β-D-xylopyranoside (for β-xylosidase). The reaction mixture contained 0.3 ml of a substrate solution (5 mM) and 0.2 mL of a 1:10 (v/v) dilution of the crop, ileum or caeca samples in 100mM phosphate buffer (pH 7.0) after centrifugation at 7211 *g* for 15 min. Incubation was carried out at 39°C and ρ-nitrophenol was quantified spectrophotometrically at A_400_ nm and at A_420_ nm (*ο*-nitrophenol concentration) after the addition of 2.5 ml of 0.25 M-cold sodium carbonate. The enzymatic activity was expressed as μmol product formed per hour per g of digesta. The above outlined procedure determines the activities of extracellular bacterial enzymes released from bacterial cells into the digesta. 

### Microbial Community Analysis by Fluorescent In Situ Hybridization (FISH)

For FISH analysis, 100 µL of the ileum digesta were diluted in PBS and pipetted onto 0.22 µm polycarbonate filters (Frisenette K02BP02500) and vacuumed (Vaccum KNF Vacuport-Neuberg). After vacuuming, the filters were transferred onto cellulose discs for dehydration in an ethanol series (50, 80, and 96%, 3 min. each). For each sample, a series of identical filters was prepared to allow the determination of optimal hybridization [18,19]. The oligonucleotides probes used for this study (Table 2) were selected from the literature. Hybridizations were carried out in 50 µL of hybridization buffer (0.9 M NaCl; 20 mM Tris/HCl, pH 7.2; 0.01% SDS) containing the oligonucleotides probes (Table 2). After hybridization, the filters were washed with washing buffer (20 mM Tris/HCl, pH 7.2; 0.01% SDS; 5 mM EDTA) for 20 min. at 48°C. The filters were rinsed gently in distilled water, air-dried, and mounted on object glasses with VectaShield (Vector laboratories nr. H-1000) anti fading agent containing DAPI (4',6-diamidino-2-phenylindole). To distinguish total count (DAPI) of bacteria from other particles in the ileum samples filters were left in 4°C for one hour in the dark until visualized using a Carl Zeiss Microscope Axio Imager M2.

**Table 2:** Oligonucleotide probes.

Target	Probe	Sequence (5' to 3')
*Bacteroides* – *Prevotella* cluster	Bac303	CCAATGTGGGGGACCTT **^*1*^**
*Clostridium perfringens*	Cperf191	GTAGTAAGTTGGTTTCCTCG2
*Enterobacteriaceae*	Enter1432	CTTTTGCAACCCACT3
*Lactobacillus* sp.*/Enterococcus* sp.	Lab158	GGTATTAGCAYCTGTTTCCA **^*4*^**

***^1^*** [19]^2,3^, [50], ***^4^*** [51]

### Statistical analysis

The data were treated statistically using one-way analysis of variance, and significance of differences between groups was determined by the Duncan’s multiple range test at the significance level of *P* equal to or less than 0.05. The calculations were tested using the GLM procedure of SAS software [20].

## Results

### Microbial community analysis

The total number of bacteria (DAPI counts) was lowest in the PC group (Table 3). Compared to the NC group, nisin did not affect the total bacterial counts. None of the dietary treatments affected the *Clostridium perfringens* and LAB counts. *Bacteroides* and *Enterobacteriacae* counts, on the other hand, were influenced by all dietary treatments. The highest counts were observed in the NC group, whereas significant reductions were observed with salinomycin and nisin supplementation; for nisin in a dose-dependent manner. 

**Table 3:** Selected microbial counts (log cfu/ml digesta) in ileal digesta determined by DAPI staining and fluorescent in situ hybridization (FISH).

	PC	NC	NI_100_	NI_300_	NI_900_	NI_2700_	SEM	*P*
DAPI	9.94**^*c*^**	10.01^ab^	10.00**^*b*^**	10.04**^*a*^**	10.01^ab^	10.00**^*b*^**	0.01	<0.001
*Bacteroides-Prevotella*	7.87**^*c*^**	8.19**^*a*^**	8.13^ab^	7.96^bc^	7.85**^*c*^**	7.82**^*c*^**	0.13	<0.001
*Clostridium perfringens*	7.77	7.71	7.87	7.82	7.82	7.70	0.13	0.369
*Enterobacteriacae*	8.01**^*b*^**	8.27**^*a*^**	7.93^bc^	7.76**^*c*^**	7.97^bc^	7.96^bc^	0.16	0.002
*Lactobacillus/Enterococcus*	8.03	8.15	8.25	8.21	8.05	8.07	0.12	0.076

PC - positive control (salinomycin, 60 mg/kg); NC - negative control (any additives);NI_100_ - 100 IU nisin/g (3.2 ml/kg); NI_300_ - 300 IU nisin/g (9.7 ml/kg); NI_900_ - 900 IU nisin/g (20.1 ml/kg); NI_2700_ - 2700 IU nisin/g (87.2 ml/kg)

DAPI - total number of bacteria determined by 4',6-diamidino-2-phenylindole staining

SEM - standard error of the mean

Within the same row, different superscripts indicate significant differences between treatments (*P*≤ 0.05)

### Microbial fermentation patterns and enzyme activities

**Figure 1:**
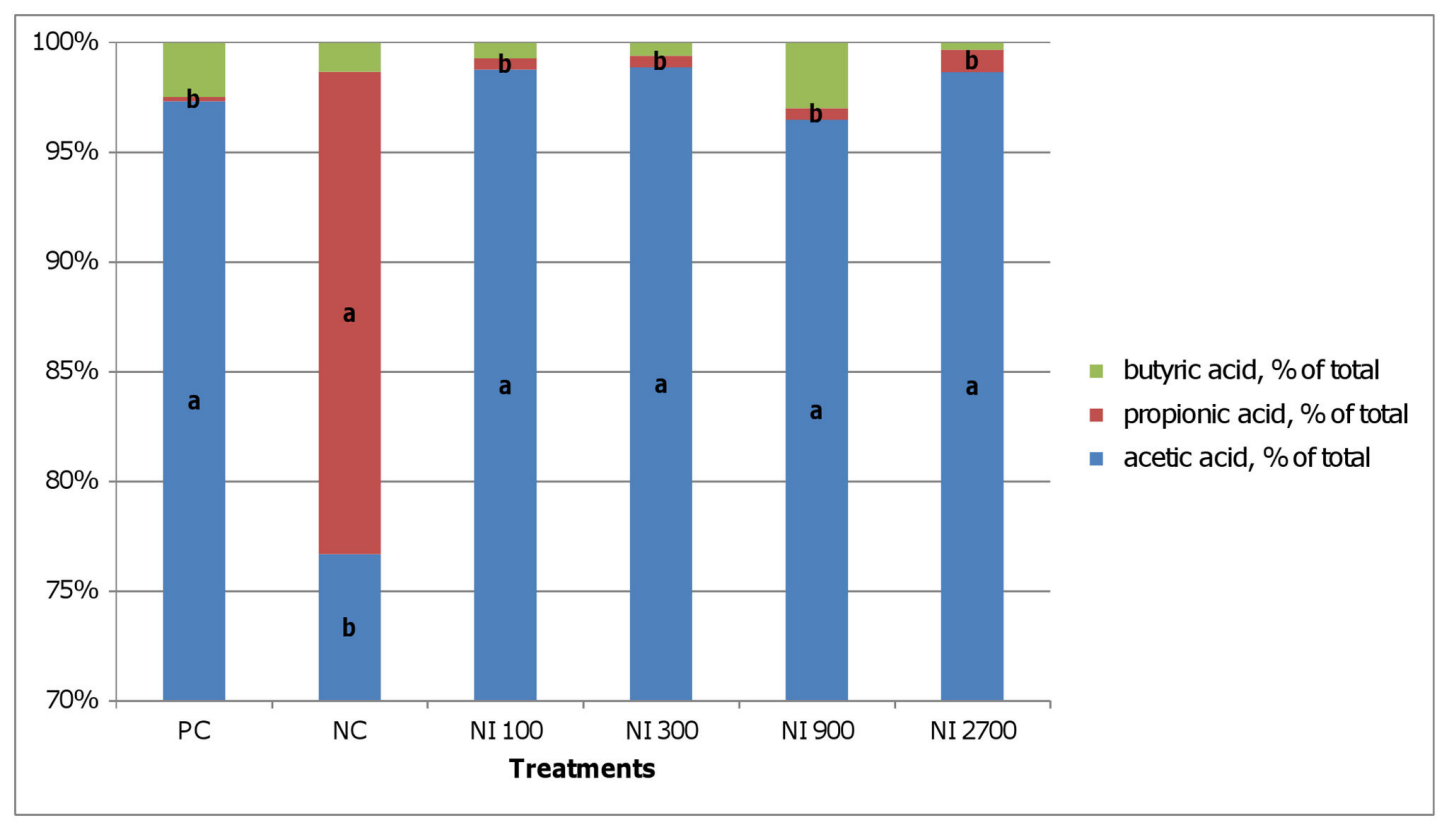
SCFA profiles in crop digesta. PC - positive control (salinomycin, 60 mg/kg); NC - negative control (any additives); NI_100_ - 100 IU nisin/g (3.2 ml/kg); NI_300_ - 300 IU nisin/g (9.7 ml/kg); NI_900_ - 900 IU nisin/g (20.1 ml/kg); NI_2700_ - 2700 IU nisin/g (87.2 ml/kg); Different letters indicate significant differences between treatments (*P*≤ 0.05).

**Figure 2:**
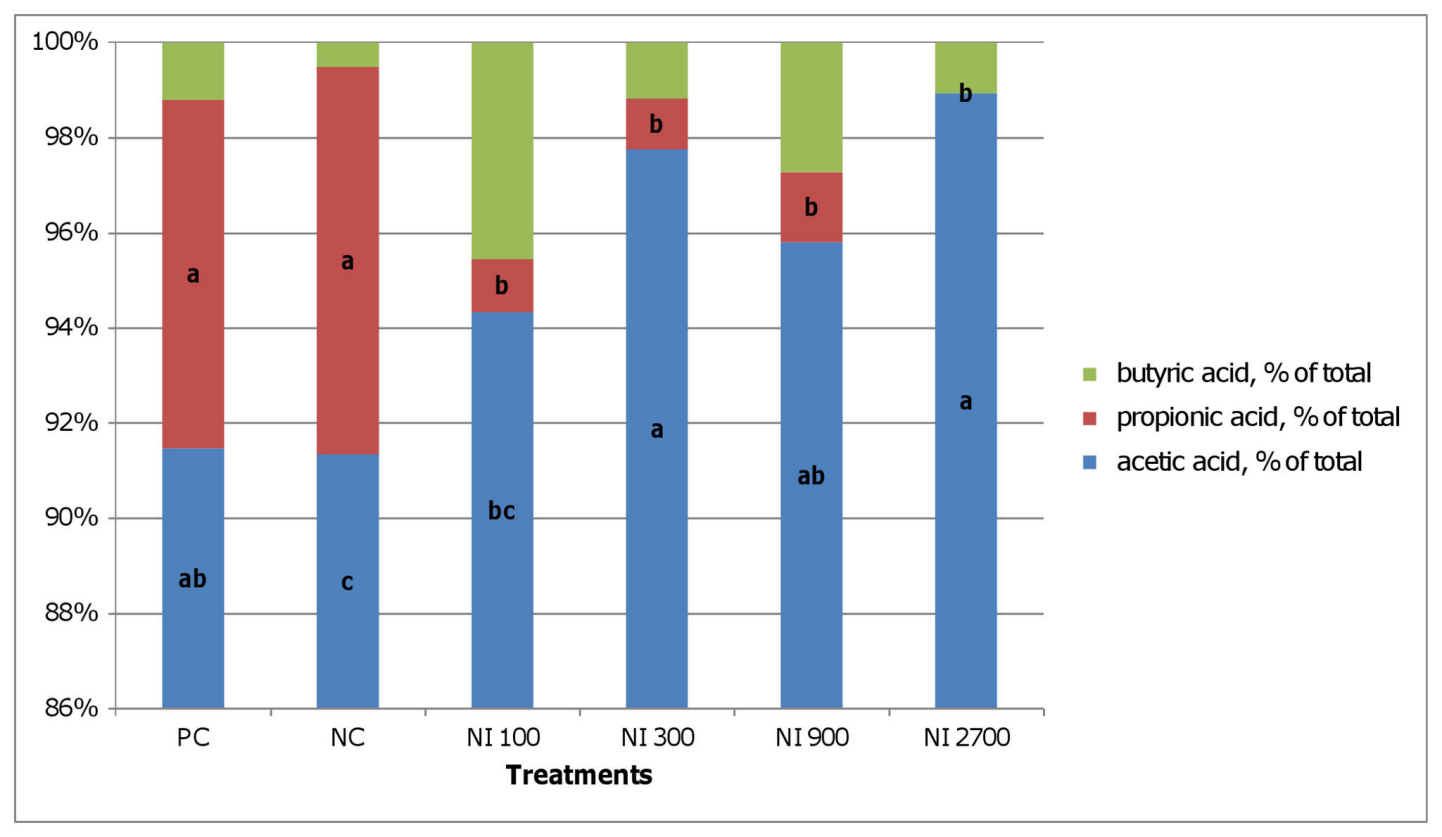
SCFA profiles in ileum digesta. PC - positive control (salinomycin, 60 mg/kg); NC- negative control (any additives); NI_100_ - 100 IU nisin/g (3.2 ml/kg); NI_300_ - 300 IU nisin/g (9.7 ml/kg); NI_900_ - 900 IU nisin/g (20.1 ml/kg); NI_2700_ - 2700 IU nisin/g (87.2 ml/kg); Different letters indicate significant differences between treatments (*P*≤ 0.05).

**Figure 3:**
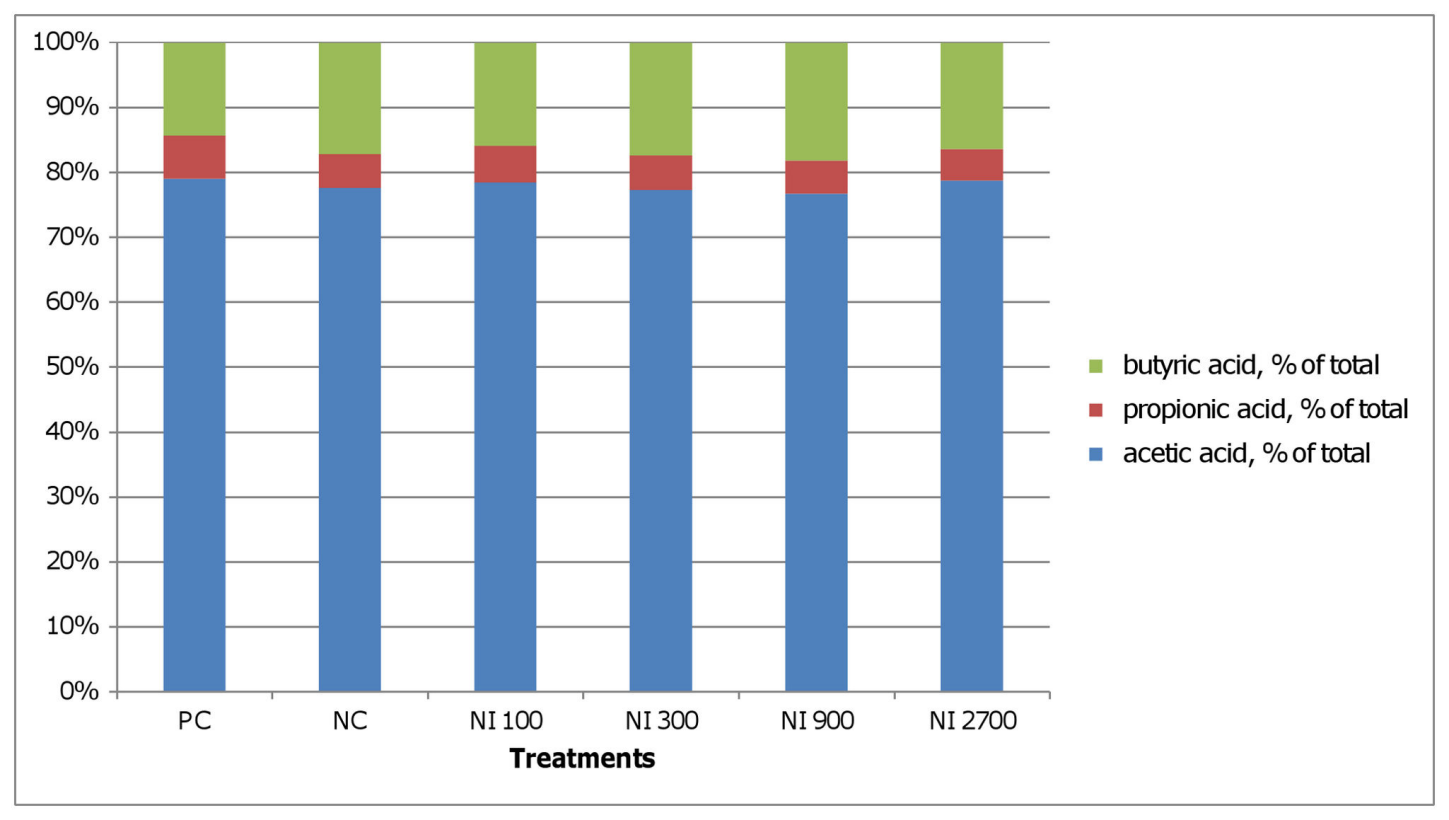
SCFA profiles in caeca digesta. PC - positive control (salinomycin, 60 mg/kg); NC- negative control (any additives); NI_100_ - 100 IU nisin/g (3.2 ml/kg); NI_300_ - 300 IU nisin/g (9.7 ml/kg); NI_900_ - 900 IU nisin/g (20.1 ml/kg); NI_2700_ - 2700 IU nisin/g (87.2 ml/kg); Different letters indicate significant differences between treatments (*P*≤ 0.05).

The microbial fermentation pattern in the broiler chicken GIT was affected by nisin and salinomycin in all examined segments (Figure 1-3, Table 4). In the crop, both additives significantly lowered the total SCFA level and dramatically changed the profile, particularly by reducing the level and proportion of propionate (Figure 1, Table 4). A similar trend was observed in ileum, however, statistically lower SCFA and PSCFA levels were observed only in the NI_300_, NI_900_ and NI_2700_ groups in comparison to the NC group (Table 4). Again, nisin reduced the proportion of propionate, whereas salinomycin did not exert this effect in ileum (Figure 2). Compared to crop and ileum, the highest total and individual SCFA and PSCFA levels were observed in the caeca digesta (Table 4). The lowest levels of acetic and propionic acids were detected with the highest dietary nisin levels. Iso-butyric acid was increased only by the salinomycin supplementation. Butyric acid concentration did not differ (*P*<0.05) between PC, NI_100,_ and NI_2700_ treatments. The highest concentration of iso-valeric acid was found in the PC group but it was only statistically different (*P*<0.05) from the NI_2700_ group. Lowest valeric acid concentration was observed in NI_2700_ however only in case of NI_300_ it was statistically different (*P*<0.05). The highest PSCFA were detected in PC while lowest in NI_2700_ (*P*<0.05), lowest total SCFA in NI_2700_ (*P*<0.05). In the caeca, nisin and salinomycin supplementation did not affect the SCFA profile (Figure 3).

**Table 4:** SCFA concentrations in crop, ileum and caeca digesta (µmol/g digesta).

	PC	NC	NI_100_	NI_300_	NI_900_	NI_2700_	SEM	*P*
Crop								
acetic acid	7.34	9.54	7.27	7.56	6.92	6.56	0.39	0.055
propionic acid	0.02**^*b*^**	2.51**^*a*^**	0.04**^*b*^**	0.04**^*b*^**	0.04**^*b*^**	0.07**^*b*^**	0.17	<.001
iso-butyric acid	0.16	0.16	0.13	0.10	0.16	0.15	0.02	0.361
butyric acid	0.18	0.14	0.05	0.04	0.12	0.02	0.02	0.065
iso-valeric acid	0.01	0.05	0.00	0.00	0.02	0.00	0.01	0.097
valeric acid	0.01	0.01	0.01	0.00	0.01	0.10	0.01	0.121
PSCFA sum	0.19	0.23	0.14	0.10	0.19	0.24	0.06	0.156
total SCFA	7.72**^*b*^**	12.4**^*a*^**	7.50**^*b*^**	7.74**^*b*^**	7.26**^*b*^**	6.89**^*b*^**	0.47	<.001
Ileum								
acetic acid	4.61	4.39	4.11	4.04	3.80	4.16	0.17	0.263
propionic acid	0.38**^*a*^**	0.33**^*a*^**	0.04**^*b*^**	0.05**^*b*^**	0.06**^*b*^**	0.00**^*b*^**	0.04	0.001
iso-butyric acid	0.38**^*b*^**	1.38**^*a*^**	1.03**^*a*^**	0.25**^*b*^**	0.44**^*b*^**	0.21**^*b*^**	0.10	<.001
butyric acid	0.05	0.03	0.25	0.05	0.11	0.05	0.03	0.107
iso-valeric acid	0.02	0.00	0.05	0.02	0.04	0.04	0.01	0.339
valeric acid	0.01	0.02	0.03	0.01	0.01	0.05	0.01	0.273
PSCFA sum	0.41**^*b*^**	1.40**^*a*^**	1.11**^*a*^**	0.29**^*b*^**	0.50**^*b*^**	0.30**^*b*^**	0.10	<.001
total SCFA	5.46^ab^	6.15**^*a*^**	5.51^ab^	4.43**^*b*^**	4.47**^*b*^**	4.51**^*b*^**	0.22	0.043
Caeca								
acetic acid	53.2^ab^	61.0**^*a*^**	59.1**^*a*^**	59.3**^*a*^**	57.2**^*a*^**	48.0**^*b*^**	1.32	0.006
propionic acid	4.38**^*a*^**	4.09**^*a*^**	4.25**^*a*^**	4.10**^*a*^**	3.82^ab^	2.86**^*b*^**	0.17	0.016
iso-butyric acid	0.69**^*a*^**	0.41**^*b*^**	0.44**^*b*^**	0.40**^*b*^**	0.45**^*b*^**	0.38**^*b*^**	0.03	0.012
butyric acid	9.69**^*b*^**	13.5**^*a*^**	12.1^ab^	13.3**^*a*^**	13.6**^*a*^**	10.0**^*b*^**	0.47	0.020
iso-valeric acid	0.48**^*a*^**	0.29^ab^	0.38^ab^	0.36^ab^	0.42^ab^	0.21**^*b*^**	0.03	0.010
valeric acid	0.68^ab^	0.65^ab^	0.71^ab^	0.81**^*a*^**	0.70^ab^	0.48**^*b*^**	0.04	0.038
PSCFA sum	1.85**^*a*^**	1.35^ab^	1.54^ab^	1.58^ab^	1.57^ab^	1.06**^*b*^**	0.08	0.012
total SCFA	69.2^ab^	80.0**^*a*^**	76.9**^*a*^**	78.3**^*a*^**	76.2**^*a*^**	61.9**^*b*^**	1.72	0.002

PC - positive control (salinomycin, 60 mg/kg); NC - negative control (any additives);NI_100_ - 100 IU nisin/g (3.2 ml/kg); NI_300_ - 300 IU nisin/g (9.7 ml/kg); NI_900_ - 900 IU nisin/g (20.1 ml/kg); NI_2700_ - 2700 IU nisin/g (87.2 ml/kg)

PSCFA - putrefactive SCFA (C4i+C5i+C5)

SEM - standard error of the mean

Within the same row, different superscripts indicate significant differences between treatments (*P*≤ 0.05)

There was no effect of treatment on crop digesta pH (Table 5). Significant pH effects were observed in ileum and caeca digesta; the highest values were observed in the PC group and the lowest values in the NI groups, but without a clear dose-dependent pattern (Table 5).

**Table 5:** pH in crop, ileum and caeca digesta.

	PC	NC	NI_100_	NI_300_	NI_900_	NI_2700_	SEM	*P*
pH								
Crop	4.95	4.87	4.82	4.90	4.94	4.93	0.03	0.714
Ileum	5.84**^*a*^**	5.72**^*a*^**	5.53^ab^	5.53^ab^	5.37**^*b*^**	5.72**^*a*^**	0.05	0.032
Caeca	6.13**^*a*^**	6.09^ab^	5.91^abc^	5.78^cd^	5.89^bcd^	5.68**^*d*^**	0.04	<0.001

PC - positive control (salinomycin, 60 mg/kg); NC - negative control (any additives);NI_100_ - 100 IU nisin/g (3.2 ml/kg); NI_300_ - 300 IU nisin/g (9.7 ml/kg); NI_900_ - 900 IU nisin/g (20.1 ml/kg); NI_2700_ - 2700 IU nisin/g (87.2 ml/kg)

SEM - standard error of the mean

Within the same row, different superscripts indicate significant differences between treatments (*P*≤ 0.05)

In general, the dietary nisin and salinomycin supplementations significantly affected the activity of specific bacterial enzymes in crop, ileum and caeca (Table 6). In crop digesta, the activities of α- and β-glucosidases, α- galactosidases and β-glucuronidase were highest (*P*<0.05) in the NC group, while nisin supplementation decreased these activities to levels found for the salinomycin treated PC group (Table 6). As compared to the PC and NC groups, the lowest β-glucosidases and β-glucuronidase activity values were observed with the highest nisin dose (NI_2700_ group). In ileum digesta, the highest α-glucosidases activity was observed in the PC group and the lowest in the NI_2700_ group (Table 6). Similar trends were observed for the other enzymes. Irrespective of treatment, the highest activity of the bacterial enzymes was found in caeca digesta (Table 6). In addition to activities of α- and β-glucosidases, α- galactosidases and β-glucuronidase, activities of α-arabinopyranosidase and β-xylosidase were analysed as well. As compared to the NC group, nisin supplementation (NI_300_, NI_900_, NI_2700_) significantly decreased the activity of α- and β-glucosidases, (NI_900_, NI_2700_), α- galactosidases (NI_100,_ NI_300_, NI_900_, NI_2700_); β-glucosidases (NI_2700_) β-glucuronidase (NI_100,_ NI_300_, NI_900_, NI_2700_); α-arabinopyranosidase (NI_2700_); β-xylosidase (NI_2700_). 

**Table 6:** Activity of extracellular bacterial enzymes in crop, ileum and caeca digesta (µmol/h/g digesta).

	PC	NC	NI_100_	NI_300_	NI_900_	NI_2700_	SEM	*P*
Crop								
α-glucosidase	0.47**^*b*^**	1.13**^*a*^**	0.50**^*b*^**	0.37**^*b*^**	0.31**^*b*^**	0.22**^*b*^**	0.06	<.0001
β-glucosidase	5.60**^*b*^**	7.14**^*a*^**	4.83^bc^	4.45^bc^	4.52^bc^	3.94**^*c*^**	0.23	<.0001
α-galactosidase	14.9	16.5	16.1	15.6	16.2	14.7	0.43	0.313
β-galactosidase	0.99**^*c*^**	6.33**^*a*^**	4.84**^*b*^**	4.18**^*b*^**	4.21**^*b*^**	2.26**^*c*^**	0.34	<.0001
β-glucuronidase	0.15^ab^	0.18**^*a*^**	0.13^ab^	0.04**^*b*^**	0.04**^*b*^**	0.04**^*b*^**	0.02	0.039
Ileum								
α-glucosidase	2.80**^*a*^**	1.98**^*b*^**	1.52^bc^	1.30^bc^	1.28^bc^	1.18**^*c*^**	0.13	<.0001
β-glucosidase	0.50**^*a*^**	0.22^ab^	0.29^ab^	0.13**^*b*^**	0.15**^*b*^**	0.09**^*b*^**	0.04	0.014
α-galactosidase	1.17**^*a*^**	1.10**^*a*^**	1.09**^*a*^**	1.10**^*a*^**	0.91**^*a*^**	0.31**^*b*^**	0.09	0.014
β-galactosidase	2.05**^*a*^**	1.59^ab^	1.27**^*b*^**	1.02**^*b*^**	0.95**^*b*^**	0.81**^*b*^**	0.18	0.003
β-glucuronidase	0.89**^*a*^**	0.23**^*b*^**	0.12**^*b*^**	0.13**^*b*^**	0.15**^*b*^**	0.12**^*b*^**	0.05	<.0001
Caeca								
α-glucosidase	20.1^ab^	23.2**^*a*^**	19.6^abc^	18.1^bc^	15.8^bc^	15.3**^*c*^**	0.71	0.001
β-glucosidase	4.96**^*b*^**	6.57**^*a*^**	5.26^ab^	3.90^bc^	3.44**^*c*^**	2.95**^*c*^**	0.27	<.0001
α-galactosidase	24.7^ab^	31.7**^*a*^**	21.5^bc^	22.2^bc^	16.3**^*c*^**	14.9**^*c*^**	1.33	<.0001
β-galactosidase	38.5^ab^	44.8**^*a*^**	41.9**^*a*^**	39.7^ab^	41.4**^*a*^**	26.1**^*b*^**	2.06	0.018
β-glucuronidase	23.0**^*b*^**	38.0**^*a*^**	19.7**^*b*^**	17.7**^*b*^**	14.4**^*b*^**	14.4**^*b*^**	1.91	<.0001
α-arabinopyranosidase	4.36**^*a*^**	4.31**^*a*^**	4.06**^*a*^**	4.13**^*a*^**	4.02**^*a*^**	2.37**^*b*^**	0.22	0.014
β-xylosidase	9.33**^*a*^**	9.63**^*a*^**	9.02**^*a*^**	7.48^ab^	6.90^ab^	4.90**^*b*^**	0.45	0.002

PC - positive control (salinomycin, 60 mg/kg); NC - negative control (any additives);NI_100_ - 100 IU nisin/g (3.2 ml/kg); NI_300_ - 300 IU nisin/g (9.7 ml/kg); NI_900_ - 900 IU nisin/g (20.1 ml/kg); NI_2700_ - 2700 IU nisin/g (87.2 ml/kg)

SEM - standard error of the mean

Within the same row, different superscripts indicate significant differences between treatments (*P*≤0.05)

### Bird performance

In the 1-14 d period, the lowest body weight gain (BWG) was recorded in the PC group (Table 7). Nisin supplementation improved BWG in all treatments as compared to PC, however, birds fed the lowest dosage of the bacteriocin did not differ from the NC group (*P*=0.001). In the 15-28 d period, only the NI_900_ and NI_2700_ groups were statistically different from the control groups. In the last period (29-35 d), the PC group was characterized by the highest BWG, but it was not statistically different from the NI_900_ and NI_2700_ groups. Over the entire experimental period (1-35 d), the highest BWG was observed for the nisin-supplemented NI_900_ and NI_2700_ groups. Dietary additives also affected the feed conversion ratio (FCR). In the 1-14 d period, the lowest FCR were observed in the NI_900_ and NI_2700_ groups and highest in the PC group. These differences were not continued in later growth stages; in the 29-35 d period, salinomycin improved FCR to the same extent as NI_2700_ but numerically it was the lowest value among all treatments. In the entire experimental period (1-35 d), the lowest FCR was observed in the treatments with highest inclusion of nisin (NI_900_ and NI_2700_). Nisin and well as salinomycin effected feed intake (FI) of the broiler chickens. In the 1-14 d and 15-28 d periods, the highest FI was observed in the NI_900_ and NI_2700_ groups. A similar trend was observed over the entire experimental period (1-35 d). 

**Table 7:** Broiler chicken performance.

	PC	NC	NI_100_	NI_300_	NI_900_	NI_2700_	SEM	*P*
BWG (g/bird)								
1-14d	313**^*b*^**	332**^*a*^**	332**^*a*^**	346**^*c*^**	360**^*d*^**	381**^*e*^**	3.12	<.0001
14-28d	859**^*a*^**	858**^*a*^**	899^ab^	900^ab^	932**^*b*^**	946**^*b*^**	8.16	0.003
28-35d	591**^*b*^**	539**^*a*^**	520**^*a*^**	530**^*a*^**	556^ab^	591**^*b*^**	6.85	0.002
1-35d	1763**^*a*^**	1729**^*a*^**	1751**^*a*^**	1776**^*a*^**	1847**^*b*^**	1918**^*c*^**	12.61	<.0001
FE (kg/kg)								
1-14d	1.42**^*d*^**	1.37**^*a*^**	1.37**^*a*^**	1.36**^*a*^**	1.32**^*c*^**	1.27**^*b*^**	0.01	<.0001
14-28d	1.62	1.63	1.59	1.62	1.60	1.58	0.01	0.754
28-35d	1.87**^*b*^**	2.01**^*a*^**	2.08**^*a*^**	1.99^ac^	1.97^abc^	1.89^bc^	0.02	0.001
1-35d	1.66**^*a*^**	1.70**^*a*^**	1.69**^*a*^**	1.68**^*a*^**	1.65^ab^	1.61**^*b*^**	0.01	0.003
FI (g/bird)								
1-14d	446**^*a*^**	456^ac^	455**^*a*^**	470^bc^	475**^*b*^**	483**^*b*^**	2.50	<.0001
14-28d	1388**^*a*^**	1391**^*a*^**	1425^ac^	1452^bc^	1489**^*b*^**	1493**^*b*^**	8.54	<.0001
28-35d	1101	1079	1073	1052	1090	1113	7.79	0.281
1-35d	2934**^*a*^**	2926**^*a*^**	2953^ab^	2973^ab^	3054bc	3089**^*c*^**	15.72	0.005

PC - positive control (salinomycin, 60 mg/kg); NC - negative control (any additives);NI_100_ - 100 IU nisin/g (3.2 ml/kg); NI_300_ - 300 IU nisin/g (9.7 ml/kg); NI_900_ - 900 IU nisin/g (20.1 ml/kg); NI_2700_ - 2700 IU nisin/g (87.2 ml/kg);

BWG -Body Weight Gain; FE - Feed Efficiency; FI - Feed Intake

SEM - standard error of the mean

Within the same row, different superscripts indicate significant differences between treatments (*P*≤ 0.05)

## Discussion

In general, the results of the present study demonstrate salinomycin as well as nisin to modulate the microbial ecology of the broiler GIT. The digesta concentrations of SCFAs were thus highest in the non-supplemented control group in each GIT segment, and the observed levels of total and individual SCFAs were in good agreement with our earlier studies [21,22]. Moreover, both additives changed the SCFA profiles of crop and ileum. The reduction of the fermentation activity in the GIT was generally in agreement with the observed bacterial enzyme activities. However, salinomycin and nisin showed slightly different modes of action. In ileum, α-glucosidase and β-galactosidase activities were highest in the salinomycin supplemented PC group, whereas nisin supplementation decreased these activities in a dose-dependent manner. 

In the present study, none of the applied additives influenced pH in the crop, but significant pH effects were observed in the ileum and caeca. In contrast to our earlier studies with bacteriocins [2], the observed pH values did not reflect SCFA concentrations. Particularly in the crop, marked changes in total SCFA were observed without any concomitant effects on digesta pH (Table 5). The lack of consistency may relate to the fact that lactic acid, known to have a major influence on digesta pH, was not analyzed in the present study.

Nisin is reported to exert antimicrobial activity against numerous LAB and some bacteria belonging to the genera *Staphylococcus, Micrococcus, Corynebacterium, Mycobacterium, Listeria,* Clostridium and *Bacillus* [23,24]. In human studies it has been reported that nisin is inactivated by proteolytic enzymes and therefore exerts no effect on the GIT microbiota [10,25]. Data from *in vivo* and *in vitro* studies with ruminants, on the other hand, indicates that nisin can influence rumen fermentation and methanogenesis [26-28], and nisin has been reported to exert effects similar to the ionophore monensin [29]. These observations indicate that nisin is not immediately degraded in the rumen. 

The commercially available nisin products are dry and contain 2.5% nisin, 74.4% sodium chloride and 23.8% of denatured milk solids. This composition makes them unsuitable for poultry nutrition due to high amount of NaCl. However, in the available literature there are many other nisin preparations obtained from *L. lactis* fermentation, which can have relatively high nisin activity combined with low NaCl content. In poultry feeds, some additives (e.g. enzymes) are used in liquid form, thus development of the cheap method of nisin production would be very interesting from this point of view. However, it must be emphasized that even though nisin is used worldwide for food preservation purposes, it is not allowed to be used for animal nutrition in many countries. The FAO/WHO Codex Committee on milk and milk products accepted nisin as a food additive for processed cheese at a concentration of 12.5 mg pure nisin per kilogram product [30]. In some countries (e.g. Peru and France) nisin is accepted without any limits in particular foods (processed cheese) while in other countries there are strong national legislations (e.g. Argentina, USA, Italy) on maximum dosage. Besides food applications there is some work done on its clinical potential. In humans, there were successful attempts to use nisin in the treatment of atopic dermatitis [31], stomach ulcers and colon infections for patients with immune deficiencies [32], as well as staphylococcal mastitis during lactation in women. Bartoloni et al. [33] tested metronidazole, vancomycin, and nisin against 60 toxigenic strains of *Clostridium difficile* collected from human subjects with *Clostridium difficile*-associated diarrhea and observed nisin to much more effective than applied antibiotics. However, most studies agree that due to nisin susceptibility to degradation by the digestive enzymes, it cannot play any role in the development of the endogenous GIT microbiota [10,25]. 

The present study demonstrates that dietary nisin can modulate the microbial ecology of the broiler chicken GIT in a manner similar to the ionophore cocidiostat salinomycin. In contrast to our findings, work done on human microflora-associated rats indicated that dietary nisin had no effect on the gastrointestinal microbial ecology, whereas diet supplementation with nisin-producing *Lactococcus lactis* strain CHCC5826, significantly affected the microbial community [10]. The differences in the observations could be linked with differences in the digestion processes in mammals and birds [34-36]; particularly digesta passage time and, hence, nisin exposure to different proteolytic enzymes. In rats digesta passage time is rather slow and around 10-13h [37], whereas in broiler chickens passage time is very rapid and usually do not exceed 3-5h [36]. The duration of the experiments could have had an impact as well; the rats were given in two single dosages for two days whereas in the present study, nisin was fed to the birds throughout the experimental period of 35 d. 

The growth promoting effect of salinomycin observed in the present study is in agreement with earlier observations on broiler chickens [38], ruminants [39] and pigs [40]. Many of the applied ionophore coccidiostats strongly influence the GIT microbiota by targeting not only *Eimeria* species but also certain Gram-positive bacteria and thereby reducing the numbers of e.g., *Clostridium perfringens* [41] and lactic acid bacteria, including *Lactobacillus* species [38]. Thus, the improvement of bird growth and feed utilization by salinomycin can be explained not only by direct suppression of coccidia, but also by inhibited growth and activity of pathogenic as well as commensal bacteria, alleviating the direct competition with the host for nutrients and decreasing e.g., the microbial deconjugation of bile salts [42,43], thereby improving fat digestibility [44]. Even though, salinomycin is usually observed to suppress the major groups of Gram-positive bacteria (e.g. LAB and *C. perfringens*), this is not always the case [2], however, it was not observed in the present study. This may relate to the fact that within e.g. the *Lactobacillus* genus, susceptibility to salinomycin has been observed to differ between species, and e.g. *Lactobacillus salivarius* is reported to be particularly susceptible [46]. A similar phenomenon may be expected for nisin. Depending on the composition of the LAB population, the overall suppressive effect of salinomycin as well as nisin may therefore not be similar from case to case. Concerning *C. perfringens*, the present data could indicate a similar phenomenon, namely that the probe may have covered/targeted other, less susceptible *Clostridiaceae*, which may have blurred the specific influence on *C. perfringens*. Moreover, in the present study we only analyzed the microbiota of the ileum. More dramatic changes could be expected to have occurred in the crop, where bacteriocins may be more active, since gizzard maceration and proteinase activity may impede the activity of these small peptides further down the GIT. The important role of the crop in the bacteriocins mode of action is also supported by our earlier findings [2] and the changes in the SCFA profile observed in the present study. 

After the EU ban of the antibiotic growth promoters, ionophores have therefore played an important role in reducing many types of bacterial enteritis in broiler chickens. However, a ban of ionophores as feed additives has been considered in the European Union as of 2012, but a final decision on this issue has been postponed due to an obvious lack of alternative disease control strategies. In this context, the growth promoting effects of nisin as observed for broiler chickens in the present study are very promising and could be expanded by investigations of the potential role of this peptide in *Eimeria* challenged birds. 

As mentioned above, information on broiler chicken performance as influenced by the use of dietary bacteriocins is scarce [45]. In our previous work [2-5], we have demonstrated however that dietary supplementation of the bacteriocin divercin AS7 in a liquid preparation improved broiler performance in a manner similar to the use of the ionophore cocidiostat salinomycin. Likewise, pediocin A, produced by *Pediococcus pentosaceus* FBB61, was observed to improve growth and feed utilization of broiler chickens challenged with *Clostridium perfringens* [7]. A significant reduction of *Campylobacter jejuni* has also been demonstrated in turkey poults after dietary addition of bacteriocin B602 from *Paenibacillus polymyxa* (NRRL B-30509) and bacteriocin OR7 from *Lactobacillus salivarius* NRRL B-35014 [46]. Finally, dietary albusin B (bacteriocin) of *Ruminococcus albus* 7 expressed by yeast, has been reported to increase intestinal nutrient absorption, elevate the fecal *Lactobacillus* counts and decrease the population of *Enterococcus* and *Salmonella*, and thereby improving the growth performance of broiler chickens [47].

The results of the present work demonstrate that nisin exerted a clearly modulating effect on the microbial ecology of the GIT, and even in an unprotected form, the nisin ultra-filtrate was able to improve bird performance significantly. Even though the detailed microbiological mechanisms behind the observed effects are still to be investigated for further elucidation, the present study is, to our knowledge, the first to demonstrate the dietary efficacy of this bacteriocin in broiler chickens. It is particular noteworthy that in comparison to salinomycin, dietary nisin in the NI_900_ and NI_2700_ treatments, improved broiler chicken body weight gain by 4.7 and 8.7%, respectively (*P*=0.0001). Moreover, birds from the NI_2700_ group were characterized by the best feed utilization (*P*=0.003) among all groups. This phenomenon can be explained by several modes of action, as modulation of host immunity and stimulation of response to *Eimeria*, similar to ionophores [48]. Improved cellular immune response after *Eimeria* infection, resulting in reduced fecal oocyst shedding [49] or improved nutrient absorption and utilization [5]. Unfortunately, the present study did not include analysis of *Eimeria* sp. colonization of the broiler chickens, wherefore direct effects of the nisin on this parasite could not be evaluated. 

## Conclusions

The findings of the present study suggest that for broiler chickens, dietary nisin exerts a mode of action similar to that of the ionophore salinomycin; they both improve broiler growth performance by modulating the microbial ecology of the GIT. Dietary nisin supplementation may thus be considered a novel strategy in poultry production for improving broiler chicken feed utilization and growth performance presumably by reducing microbial density and activity in the GIT.
